# Proximal tibia stress fracture with Osteoarthritis of knee − Radiological and functional analysis of one stage TKA with long stem

**DOI:** 10.1051/sicotj/2018006

**Published:** 2018-04-18

**Authors:** Dhanasekaran Soundarrajan, Natesan Rajkumar, Palanisamy Dhanasekararaja, Shanmuganathan Rajasekaran

**Affiliations:** Department of Orthopaedics, Ganga hospital, Coimbatore India

**Keywords:** Stress fracture, Proximal tibia, Osteoarthritis, TKA with long stem

## Abstract

*Introduction:* Proximal tibia stress fractures with knee osteoarthritis pose a challenging situation. We evaluated the radiological and functional outcome of one-stage total knee arthroplasty (TKA) and long stem for patients with varied grades of knee arthritis and proximal tibia stress fractures.

*Methods*: We analysed 20 patients from April 2012 to March 2017 with proximal tibia stress fractures associated with knee osteoarthritis of varied grades. Out of 20 patients, five were acute fresh fractures. The mean age was 64 years (range, 52–78) which includes three men and 17 women. Previous surgery in the same limb, rheumatoid arthritis, valgus deformity were excluded. All patients were treated with posterior stabilised TKA with long stem, of which, four patients had screw augmentation for medial tibial bone defect and two patients with malunited fracture at stress fracture site required osteotomy, plating and bone grafting. Two patients had two level stress fracture of tibia in the same leg.

*Results*: The mean follow-up period was 28 (range, 6–60) months. The mean tibiofemoral angle improved from 18.27° varus to 1.8° valgus. The mean knee society score improved from 21.9 (range, −10 to 45) to 82.8 (range, 15–99) [*p* < 0.05]. The mean Knee Society functional score improved from 15.5 (range, −10 to 40) to 76.8 (range, 10–100) [*p* < 0.05]. All fractures got united at the last follow-up. One patient had infection and wound dehiscence at six months for which debridement done and had poor functional outcome.

*Conclusion*: TKA with long stem gives excellent outcome, irrespective of severity of arthritis associated with stress fracture. By restoring limb alignment and bypassing the fracture site, it facilitates fracture healing. Early detection and prompt intervention is necessary to prevent the progression to recalcitrant non-union or malunion.

## Introduction

Fractures occurring due to repeated micro trauma on the bone over a prolonged time period are called stress fractures [1]. It can be divided into two groups, namely Fatigue and Insufficiency fractures. Normal bones when subjected to repeated abnormal stress will lead to fatigue fractures. Young athletes and military personnel's with normal bones are more prone for tibial stress fractures [2].

Normal stress on an abnormal underlying bones will cause Insufficiency fractures [3]. In elderly people, stress fractures are associated with abnormal underlying bone. The condition is commonly associated with osteoporosis. Other risk factors reported in the literature were hyperparathyroidism, rheumatoid arthritis, Paget's disease, pyrophosphate arthropathy and after total knee arthroplasty [4–9].

Osteoarthritis of knees with associated coronal deformities like varus or valgus will lead to asymmetrical loading and abnormal repeated stress concentration in the metaphyseal area of proximal tibia leading to insufficiency stress fractures [10,11]. This is compounded by underlying osteoporotic bone in these elderly patient group.

Treatment options for these dual problem of stress fracture along with osteoarthritis described in the literature include treating the stress fracture alone or to combine with osteoarthritis treatment. Cast immobilisation, knee bracing or internal fixation are advocated for treating stress fracture [12].

**Figure 1:**
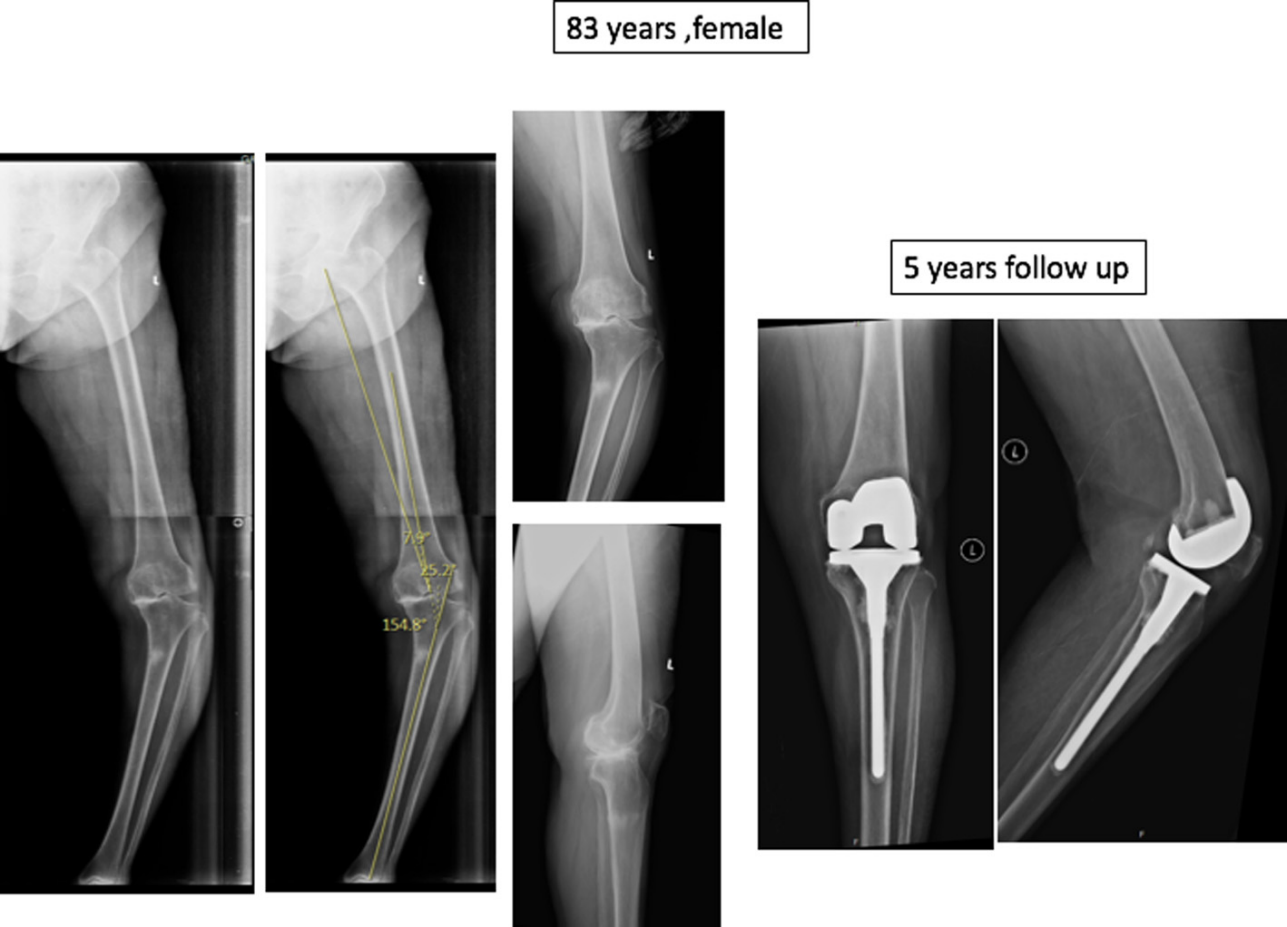
Chronic proximal tibial stress fracture treated with long stem TKA.

Total knee arthroplasty (TKA) with long stem address both the problems by arthritis correction, restoring limb alignment and bypassing the fracture site which aids in union and helps faster recovery in these elderly patient group Figure 1.     For treating deformity at stress fracture site, osteotomy and plate fixation or long stem TKA with unicortical plating were described.

**Figure 2:**
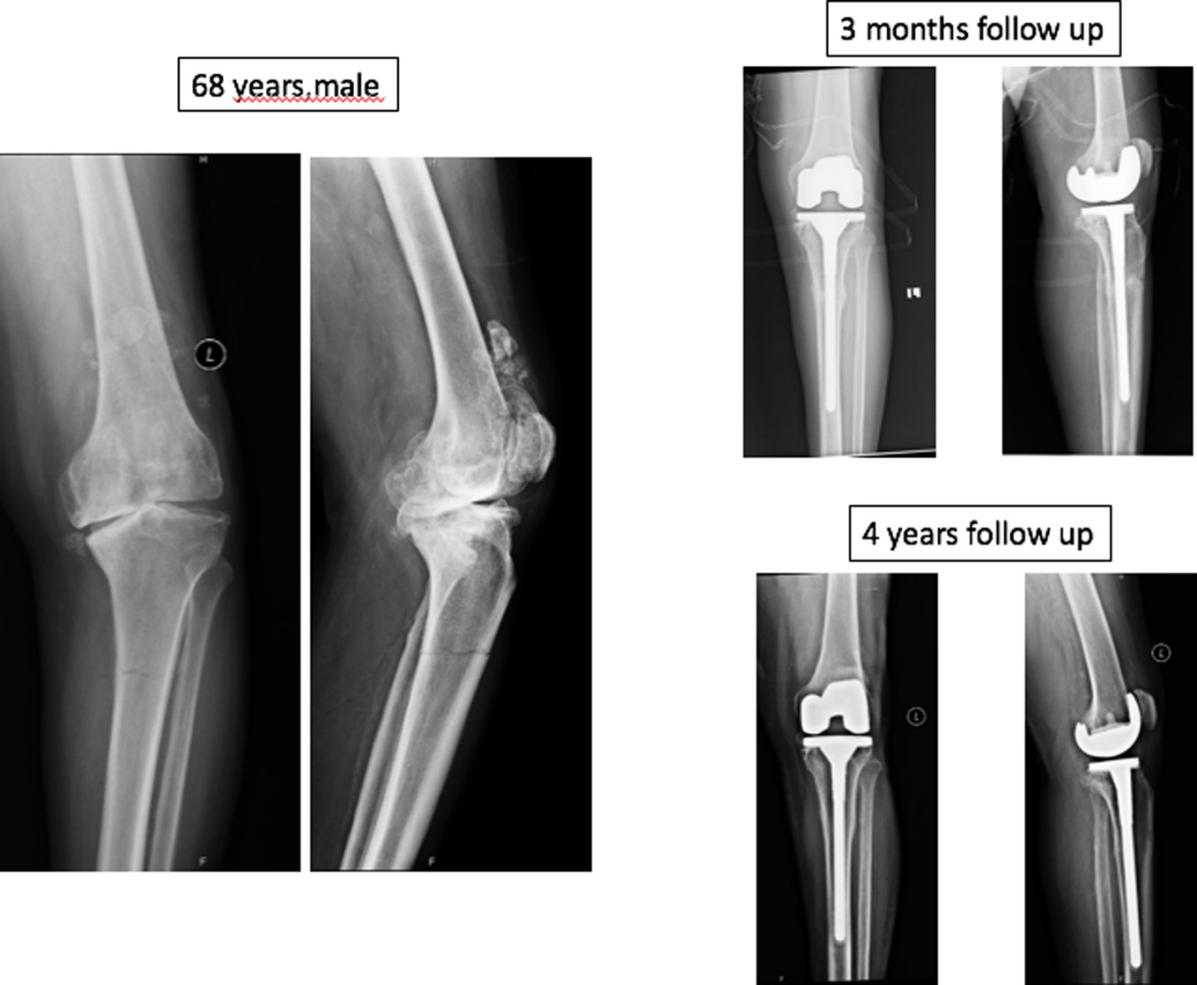
Acute stress fracture treated with long stem TKA.

Our aim is to analyse the radiological and functional outcome of stress fracture associated with osteoarthritis and varus deformity treated with one stage TKA and long stem Figure 2. We also assessed any preoperative predictive factors which may lead to stress fracture in this patient group.

## Materials and methods

Patients with proximal tibia stress fracture associated with degenerative osteoarthritis and varus deformity from April 2012 to March 2017 in our institution were analysed. Twenty patients were included in the study. Intraarticular stress fractures, Patients with rheumatoid arthritis, valgus deformity, abnormal bone disease like Pagets or severe osteoporosis are excluded from the study. All patients had unilateral stress fracture and were evaluated preoperatively using the Knee Society score and knee society functional score.

Full length alignment X rays, Anteroposterior, Lateral and skyline views of knees were taken for preoperative workup. For patients who are unable to stand, full length alignment X rays not taken, but long films taken to cover metadiaphyseal area on either side of the affected knee. The amount of varus deformity quantified by tibiofemoral angle and graded into mild (less than 10 degrees), moderate (10–20 degrees) and severe (more than 20 degrees). Medial distal femoral angle and medial proximal tibial angle were calculated. All stress fractures diagnosed by plain radiographs. On Xray, features suggestive of stress fractures include frank fracture line, periosteal bone formation, endosteal callus, and horizontal or oblique linear patterns of sclerosis [13]. Preoperative serum Vitamin D levels, calcium, phosphorous, alkaline phosphatase and paratharmone levels were recorded.

**Figure 3:**
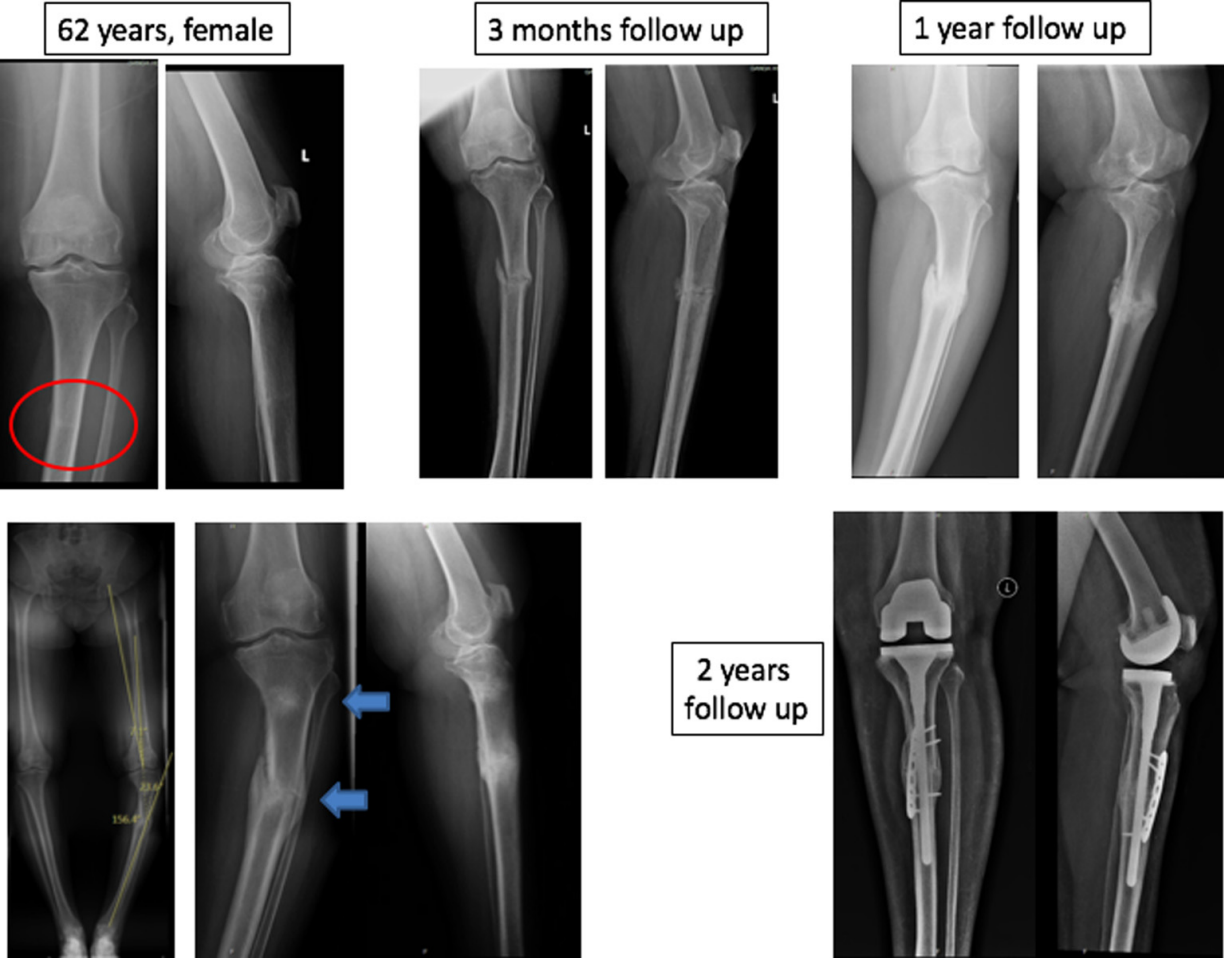
Two level stress fracture with malunion at stress fracture site treated with TKA with long stem augmented with plating and bone grafting.

There were five acute (fracture line visible) and fifteen chronic fractures (endosteal callus and sclerosis at the proximal tibial level) based on the radiographs at presentation. No patients had any trauma or fall. All patients were treated with posterior stabilised TKA with long stem, of which, four patients had screw augmentation for medial tibial bone defect. Two patients initially treated with cast application which went into malunion at stress fracture site required osteotomy, plating and bone grafting Figure 3. Two patients presented with Nonunion at stress fracture site also treated with TKA with long stem.

Through standard midline approach and medial parapatellar arthrotomy, posterior stabilised TKA (19 cases −PFC, Depuy and one case −Genesis II, Smith and Nephew) done. Tibial cuts taken using extramedullary jigs. After placing the tibial tray, the entry point for the tibial stem taken and serial reaming done and fracture site bypassed using long tibial stem. The stem bypassing the fracture site acted as an internal splint and stabilised the fracture. The length of the stem decided peroperatively bypassing the fracture site and confirmed with fluoroscopy. On cementing the components, care was taken to avoid the intrusion of cement into the fracture site and only the base plate was cemented.

**Figure 4:**
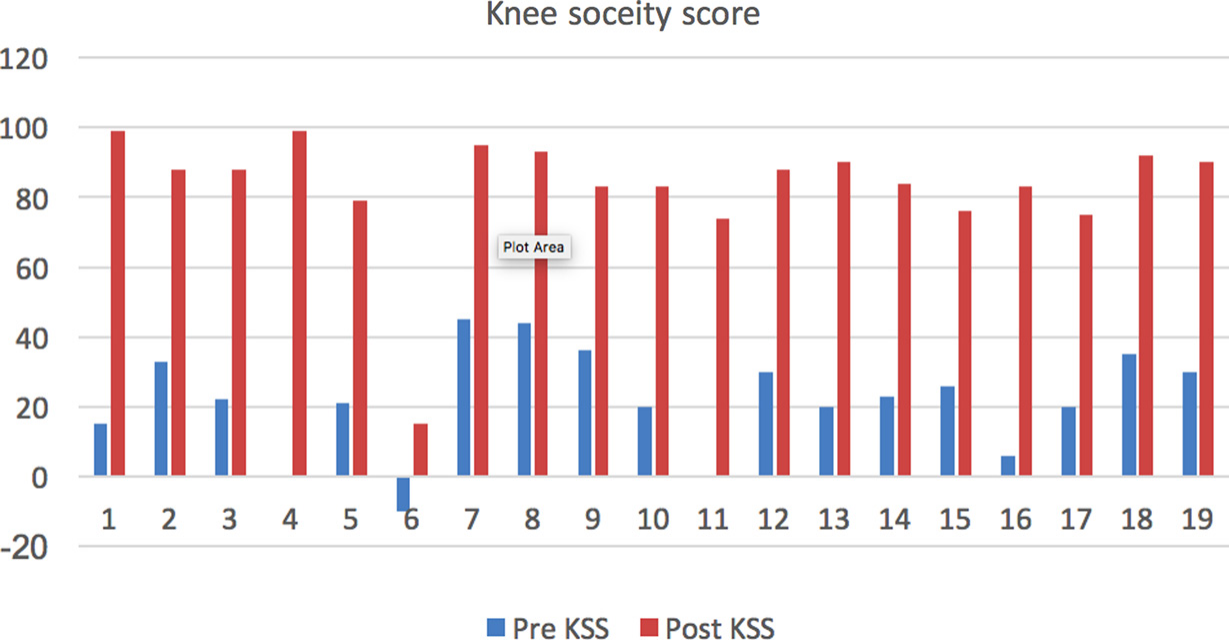
Knee society score – Pre and Postop.

Partial weight bearing walking with walker support for four weeks and full weight bearing allowed after four weeks for eighteen patients. For two patients who underwent augmentation plating, six weeks of partial weight bearing allowed and then switched over to full weight bearing mobilisation. Patients were followed up at months 1, 3, 6, and 12, and yearly thereafter. Radiographs taken during followup to examine for bony union. All patients were evaluated using knee society score, knee society functional score and radiologically assessed using full length radiographs Figure 4.

### Statistical analysis

**Figure 5:**
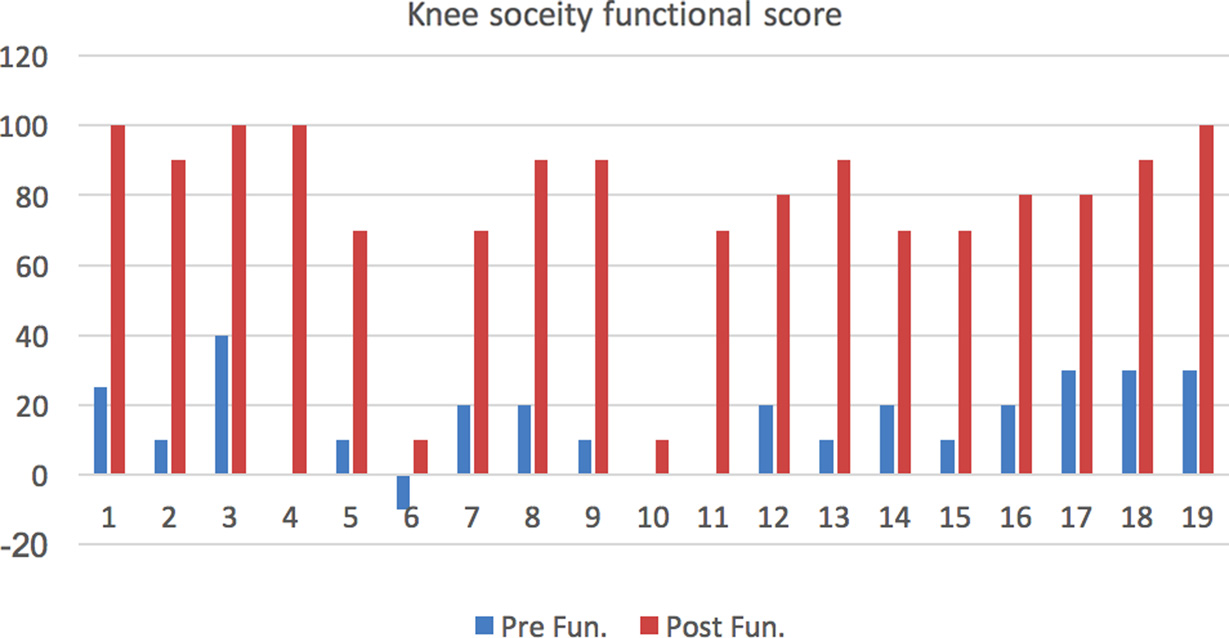
Knee society functional score − Pre and Postop.

The data were analysed using SPSS for Windows Inc. Version 19 (Chicago, Illinois). Descriptive statistics were presented as Mean (SD) for continuous variables. Preoperative Knee Society scores and the functional score was compared with postoperative KSS and functional scores using the paired t-test to compare the means between two related groups on the same continuous, dependent variable Figure 5. A two-sided *p*-value (*p* < 0.05) was taken as statistically significant. Multiple bar diagram was used to represent two sets of inter-related data.

## Results

The mean age was 64 years (range, 52–78) which includes three men and 17 women. Eleven patients had involvement in right proximal tibia and nine had left sided stress fractures. The average BMI is 31.2 (21.6–42.0).The mean follow-up period was 28 (range, 6–60) months. Five patients had mild varus (less than 10 degrees), six patients had moderate varus (10–20 degrees) and Nine patients had severe varus (more than 20 degrees). The mean preoperative tibiofemoral angle is 18.27° varus. The mean deformity at the malunited fracture site in the two patients is 14.6 degrees. The mean Medial distal femoral angle was 93.4 and mean medial proximal tibial angle was 83.8. Two patients had two level stress fracture of tibia of the same leg. Eighteen patients had bilateral osteoarthritis of knees of which 12 patients underwent total knee arthroplasty of the next knee within 4 months. One patient died at final followup leaving nineteen patients for functional analysis.

The preoperative Vitamin D level is 23.8 ng/mL. Metabolic disease workup with calcium, phosphorous, alkaline phosphatase and parathormone found within normal limits in all patients. The mean tibiofemoral angle improved from 16.57° varus to 1.8° valgus. The mean knee society score improved from 21.9 (range, −10 to 45) to 82.8 (range, 15–99) [*p* < 0.05].The mean Knee Society functional score improved from 15.5 (range, −10 to 40) to 76.8 (range, 10–100) [*p* < 0.05]. All fractures were united at the four to six months follow-up. One patient had infection and wound dehiscence at six months for which debridement done and had poor functional outcome.

## Discussion

Young people, especially athletes are prone for stress fractures and commonly reported sites include tibial shaft, femoral neck, metatarsal neck and fibula. Of which, incidence is more common in shaft of tibia, which accounts for more than 50% of fractures [14]. Stress fracture in the elderly people are reported in association with abnormal underlying bone like osteoporosis. The other conditions reported are rheumatoid arthritis, pyrophosphate arthropathy, Pagets disease [4–8] and after knee arthroplasty (unicondylar and navigated) [15–17].

In osteoarthritis, coronal deformities like varus or valgus deformity shift the mechanical axis away from the knee and load in the proximal tibia become uneven which leads to abnormal repetitive stress concentration in the metaphyseal area of proximal tibia and this stress also compounded by sagittal deformities in moderate and severe arthritis [10]. The critical biomechanical factor involved in the etiology and healing of these stress fractures is determined by the coronal plane alignment [11].

There is time delay in diagnosing the stress fracture and the various reasons quoted in the literatures are lack of suspicion, ignoring pain and tenderness over the proximal tibia, sudden worsening of arthritis attributed to degenerative meniscal tear or dislodged loose body and not ordering full length radiographs [12]. The first significant symptom of the stress fracture should be recognised and promptly addressed, since the delay in instituting treatment will lead to progression and affect the healing pattern of stress fracture due to malalignment associated with coexisting osteoarthritis.

The diagnosis of stress fracture in most of the patients described in the literature are by plain radiographs [12]. We diagnosed all patients with the help of radiographs. In doubtful scenario, Magnetic resonance scan and bone scan may help in the diagnosis [18]. Sudden worsening in severe osteoarthritis patient with pain over proximal tibia should arouse the suspicion of stress fracture and full length radiographs and serial radiographs are necessary to detect the stress fracture.

Even without severe osteoarthritis, stress fracture may occur. In our series, eleven patients had mild to moderate osteoarthritis (less than 20 degrees), but presented with stress fracture. In these patient group, treating stress fracture alone will lead to persistent malalignment at knee level and may lead to non-union and progression of deformity. Two stage surgery will also lead to prolonged treatment and increase the morbidity in these elderly patient group.

Serum calcium, phosphorous, alkaline phosphatase, parathormone help to ruleout any underlying metabolic bone disease. All our patients had metabolic parameters within their normal levels. 17 patients had low serum Vitamin D levels (less than 30 ng/mL). Female patients are far more compared to the male patients in our series. We believe Low vitamin D level with female sex and concomitant osteoporosis predispose to proximal tibia stress fracture along with osteoarthritis. We have not checked Bone mineral density in our patients, to grade the amount of osteoporosis.

Two patients had two level stress fracture of tibia of the same leg. This is rare phenomenon where second stress fracture occur in the same tibia. To the best of our knowledge, it has not reported in the literature. Eighteen patients had bilateral osteoarthritis of knees of which 12 patients underwent total knee arthroplasty of the next knee within 4 months. Hence, we believe stress fracture are more common in bilateral osteoarthritis of knees with instability.

Management of these stress fractures is challenging and is either conservative or surgical. Conservative management includes Non-weight bearing alone, cast immobilisation or electrical stimulation [12]. Many cases [10,11] treated conservatively described in the literature subsequently went for non-union or pseudoarthrosis with persistent malalignment and further aggravating the symptoms of osteoarthritis which later required surgery.

Malalignment secondary to osteoarthritis increases the stress at the fracture site, which predisposes to delayed or non-union [13]. In our series, four patients initially treated with cast immobilisation subsequently went for non-union or malunion with deformity and they eventually underwent long stem TKA, of which, two patient requiring plate fixation and bone grafting. Thus conservative treatment which holds good in young patients with stress fracture may not be successful in treating patients associated with osteoarthritis with deformity.

Pseudoarthrosis described more commonly with stress fractures being diagnosed late or treated conservatively [10,12,19] and makes definitive treatment even more difficult. Moreover, prolonged cast immobilisation will be associated with morbidity in these patient age group. Long term immobilization causes knee stiffness to worsen and does not resolve the pain and symptoms caused by osteoarthritis [10]. Mullaji et al. [18] also showed in their series, that the presence of malalignment at knee level and associated stress fracture may lead to recalcitrant non-union.

The surgical options described in the literature include internal fixation with plates, osteotomy for correction of axis deformity with plate fixation and TKA at a later date, TKA and non–weightbearing until fracture healing, long-stem TKA for fracture bridging, TKA and intramedullary nailing and combined TKA with long-stem and internal fixation with plate and bone grafting for rotary stability and fracture bridging [10,12,19,20].

Two stage procedure like plating initially followed by TKA in treating these stress fracture will lead to prolonged treatment and pain due to arthritis and malalignment will be persisting till the second procedure and this is not an appealing option. One-stage procedure with long tibial stem bypassing the fracture site addresses both the problems of correcting the arthritis and malalignment and acts as an internal splint for the fracture. This also requires single anaesthesia, allows fracture healing because of its load sharing function of the stem and aids in quicker recovery [20,21].

For stress fracture associated with Nonunion and deformity, long tibial stem alone may not be sufficient. Augmentation with plate and bone grafting allows rotational stability [21] and two patients in our series with pseudoarthrosis underwent osteotomy with augmentation plating and bone grafting which eventually healed.

One patient in our series had periprosthetic joint infection and she underwent wound debridement at six months postop and she had poor functional outcome. Even for that patient, stress fracture found united at four months followup. In our series, we also included patient with six months followup, since we believe that fracture union can be visualised and functional outcome can be evaluated at this period of time.

To conclude, Proximal tibial stress fracture associated with osteoarthritis and varus deformity adds the complexity of the situation. Mild to moderate osteoarthritis also may present with stress fracture and one should not hesitate to treat with total knee arthroplasty. Early detection and intervention is the key to prevent the progression of the disease and deformity. Bilateral knee osteoarthritis, Elderly females, obesity and concomitant osteoporosis are the risk factors associated with these stress fractures. Thorough clinical and laboratory evaluation and careful preoperative planning is essential in managing this complex problem.

Conservative treatment with prolonged cast immobilisation in these patients may lead to pseudoarthrosis or non-union and morbidities associated with immobilisation. We believe, this is one of the largest series and unique, since we included only proximal tibia stress fracture along with osteoarthritis and reported two cases of two-level stress fracture of tibia of same leg. Patients treated by TKA and long stem for acute or chronic stress fractures, gives excellent outcome, irrespective of severity of arthritis. By restoring limb alignment and bypassing the fracture site, it facilitates fracture healing. Early detection and prompt intervention is necessary to prevent the progression to recalcitrant non-union or malunion.

## Conflict of interest

The authors declare that they have no conflicts of interest in relation to this article.
